# The cyclic expansion and contraction characteristics of a loess slope and implications for slope stability

**DOI:** 10.1038/s41598-021-81821-4

**Published:** 2021-01-26

**Authors:** Hengxing Lan, Xiaoxia Zhao, Renato Macciotta, Jianbing Peng, Langping Li, Yuming Wu, Yanbo Zhu, Xin Liu, Ning Zhang, Shijie Liu, Chenghu Zhou, John J. Clague

**Affiliations:** 1 LREIS, Institute of Geographic Sciences and Natural Resources Research, Chinese Academy of Sciences, Datun Road 11A, Beijing, 100101 China; 2 School of Geological Engineering and Geomatics, Chang’an University, Xi’an, 710054 China; 3 University of Chinese Academy of Sciences, Beijing, 100049 China; 4 Department of Civil and Environmental Engineering, University of Alberta, 6-207, 9211 116, St. Edmonton, AB T6G 1H9 Canada; 5 Department of Earth Sciences, Simon Fraser University, 8888 University Drive, Burnaby, V5A 1S6 Canada

**Keywords:** Natural hazards, Solid Earth sciences, Geology, Engineering, Civil engineering

## Abstract

Loess covers approximately 6.6% of China and forms thick extensive deposits in the northern and northwestern parts of the country. Natural erosional processes and human modification of thick loess deposits have produced abundant, potentially unstable steep slopes in this region. Slope deformation monitoring aimed at evaluating the mechanical behavior of a loess slope has shown a cyclic pattern of contraction and expansion. Such cyclic strain change on the slope materials can damage the loess and contribute to slope instability. The site showing this behavior is a 70-m high loess slope near Yan’an city in Shanxi Province, northwest China. A Ground-Based Synthetic Aperture Radar (GB-SAR) sensor and a displacement meter were used to monitor this cyclic deformation of the slope over a one-year period from September 2018 to August 2019. It is postulated that this cyclic behavior corresponds to thermal and moisture fluctuations, following energy conservation laws. To investigate the validity of this mechanism, physical models of soil temperature and moisture measured by hygrothermographs were used to simulate the observed cyclic deformations. We found good correlations between the models based on the proposed mechanism and the exhibited daily and annual cyclic contraction and expansion. The slope absorbed energy from the time of maximum contraction to the time of maximum expansion, and released energy from the time of maximum expansion to the time of maximum contraction. Recoverable cyclic deformations suggest stresses in the loess are within the elastic range, and non-recoverable cyclic deformations suggest damage of the loess material (breakage of bonds between soil grains), which could lead to instability. Based on these observations and the models, we developed a quantitative relationship between weather cycles and thermal deformation of the slope. Given the current climate change projections of temperature increases of up to 3.5 °C by 2100, the model estimates the loess slope to expand about 0.35 mm in average, which would be in addition to the current cyclic “breathing” behavior experienced by the slope.

## Introduction

Loess is widely distributed in North and Northwest China, covering approximately 6.6% (630,000 km^2^) of the country’s land surface^1,2^. These deposits are fertile and have played an important role in the development of the Chinese economy^3,4^. Natural processes and widespread development in these areas have left steep slopes^5^ that are potentially unstable and of public concern. Therefore, understanding the mechanisms leading to instability in these slopes is critical to the sustainability of the economic activities in these areas.

Cyclic expansion and contraction of loess slopes due to thermal heating and cooling is an important process with the potential to degrade the stability of these slopes that has not received much attention in the past. Loess comprises mineral grains, mainly silt-size, commonly with cementing agents providing some bonding between the grains. Cyclic temperature changes in soils can cause thermal shock and fatigue (due to associated cyclic changes in stresses), causing progressive damage of the loess material (progressive break of the bonds between soil grains) and ultimately lead to mechanical failure^6,7^. Although the influence of thermal changes is most pronounced near the surface, progressive damage can occur in large areas of the slopes. With time, this generates a surficial layer of damaged material that is susceptible to instability. When a portion of this layer fails, the falling debris tends to cause instability of the material downslope, and the scarp of the initiating failure tends to destabilize material upslope. Therefore, shallow failures have the potential for large volume runouts. Enhanced understanding of the fundamental mechanism of cyclic deformation in loess slopes can therefore lead to improved monitoring strategies for landslide risk management in loess regions.

In this paper, we use the term “soil” in an engineering sense to refer to unconsolidated and poorly consolidated surface and near-surface earth materials, including loess. Thermal shock occurs when temperature changes within the soil mass are sufficiently large that the material cannot accommodate the resulting strains without suffering damage. This damage represents a reduction in soil strength, as bond breakage reduces the cohesive component of strength. Commonly, deformation caused by temperature changes is not large enough to cause significant damage, however fatigue caused by the cyclic behavior progressively weakens the strength of the soil. In the long term, this process can lead to degradation of the soil strength to a point of imminent failure^8^.

The effect of temperature-induced deformations has been previously studied, with examples of thermal cyclic deformations and fatigue including the annual and daily displacements of tall engineered structures^9^, rock slopes^10^, embankments^11^, as well as rock fall processes triggered by cyclic thermal stresses acting on exfoliation fractures^12^. However, the authors are not aware of work attempting to quantify the relationship between thermal fluctuation, deformations in loess slopes, and their effects on potential slope failures.

In order to gain insight into the relationship between weather and slope deformation, robust slope monitoring strategies are required to provide accurate, repeatable, and frequent information. Traditional geodetic techniques for landslide monitoring include leveling^13^ and GPS (Global Positioning System)^14,15^. Although these techniques can provide highly accurate deformation measurements, the measurements are spatially sparse. Spaceborne InSAR (synthetic aperture radar interferometry) techniques have been widely used in surface deformation monitoring^16–19^ and provide land deformation maps in the line of sight between the satellite and the area of interest. However, their sampling frequency depends on the satellite revisit period, which can range between days and weeks depending on the satellite system used and the location of the area of interest. GB-SAR (Ground-based Synthetic Aperture Radar) has been developed during the last decade^20^ and is characterized by finer spatial resolution than satellite instruments (less than one meter compared to 3 m × 3 m at best for satellite instruments), and high frequency of data acquisition (several images per hour)^21^. GB-SAR was adopted in this study for monitoring the loess slope deformation patterns.

The temperature-dependent behavior raises the question of the potential impact of a changing climate in the amount of strain imposed to loess slopes. During the twentieth century, Earth’s surface temperature increased by approximately 0.6 °C, and additional warming is anticipated through the present century^22^. Discussion on the impacts of global warming have focused on glacier and ice sheet melting, sea-level rise, and extreme weather events, such as cyclones, floods, and droughts; and less so on the effects of local and regional increases in landslide frequency, in particular the stability of loess slopes. Understanding the potential effects of climate change in the stability of loess slopes becomes increasingly important for sustainable development in these areas.

**Figure 1:**
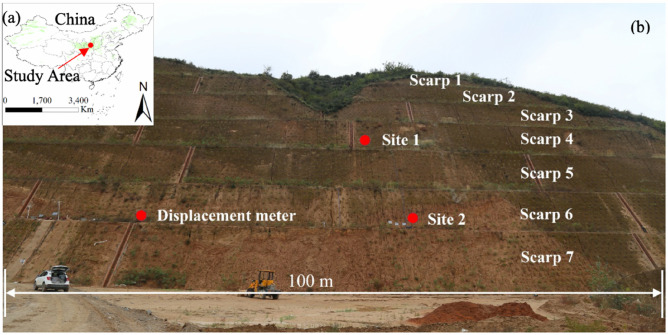
(**a**) Location of the loess slope and the distribution of loess and loess-like soils (green shading). (**b**) View of the loess slope near Yan’an. The red dots indicate the locations of instruments.

**Figure 2:**
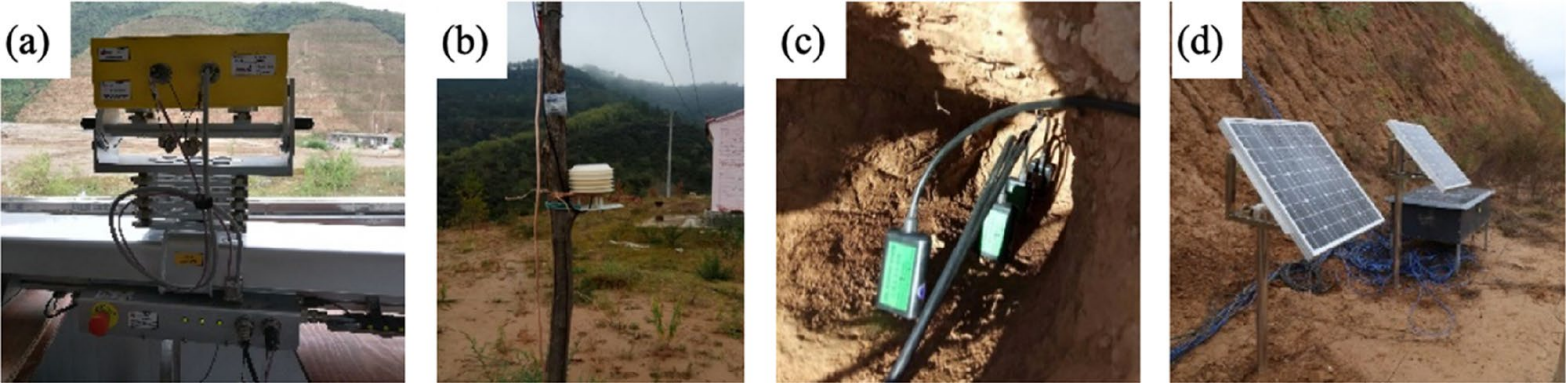
(**a**) GB-SAR instrument facing the slope. (**b**) Weather station for collecting meteorological data, installed on a pole approximately 2 m from the GB-SAR instrument and 1.5 m above the ground surface. (**c**) Hygrothermograph for collecting soil temperature and moisture data at four depths (0.1, 0.25, 0.4, and 0.6 m into the slope), installed at sites 1 and 2 (Fig. 1). The photo was taken prior to inserting the sensors into the slope. (**d**) Solar panels and the box containing the data acquisition system.

In this paper, we present the spatial–temporal characteristics of the deformation of a loess slope and show how the deformation correlates to moisture content and temperature fluctuation. The effects of changes in soil temperature and moisture on the loess slope deformation are analyzed using both empirical and physical models. We propose a mechanism that explains such behavior, validated with observations from high-frequency deformation and weather data. The mechanism is used to develop a model to estimate short term effects of weather fluctuations, and the impact of increasing temperatures due to climate change, on the short-term cyclic and long-term average expansion of the slope. The loess slope that is the subject of this work was excavated on the side of a valley slope and is approximately 70-m high (Fig. 1). The location is near the city of Yan’an, in Shanxi Province, China. One year of slope surface displacements were measured using a GB-SAR unit and a displacement meter. Soil temperature and moisture were also measured at the site (Fig. 2). The mechanisms and methods presented here can be adopted at other loess-rich areas to better understand the potential impacts of a changing climate on the deformation behavior of loess slopes and the increased potential for fatigue-induced damage and slope failure.

## Study area and data acquisition

### Study area

The study area is a loess slope near Yan’an, Shanxi Province, northwestern China (N 36°35′07″, E 109°18′39″). Yanan is located in the central part of a loess plateau, with winters that are snowless and semi-dry. Air moisture and temperature increase in the summers. The annual average temperature is 9.2 °C and the annual average precipitation is 550 mm.

The loess slope of this study is a hillside cut excavated in 2015 to increase the available land for farming. Measures to reinforce the slope, including planting vegetation and installing drainage channels, were completed by early 2017. The slope geometry consists of six benches. The elevation at the bottom is 1190 m above sea level (a.s.l.) and the slope length of 230 m. The elevation at the top of the slope is 1260 m a.s.l., and the slope length there is 150 m. The total area of the slope is approximately 13,000 m^2^. The average slope angle is 48°. During the monitoring campaign, the planted vegetation was still small and sparse, resulting in a relatively bare slope, ideal for GB-SAR monitoring. A photograph of the test site is shown in Fig. 1.

### Data acquisition

#### GB-SAR system

The GB-SAR sensor used in this study is an Image by Interferometric Survey-L (IBIS-L) system made by Ingegneria Dei Sistemi (IDS). The sensor has a Ku band antenna with a wavelength of approximately 1.72 cm and a maximum effective range of 4 km. It can provide line-of-sight (LOS) deformation information with a precision of up to 0.1 mm under ideal conditions^23^. The sensor was installed on a stable solid horizontal base, approximately 200 m across the valley from the slope. During one year, from 30 September 2018 to 30 September 2019, a dataset of 24,518 images was acquired by the IBIS-L instrument. The measurement parameters used for the campaign are summarized in Table 1. A photograph of the field set-up of the IBIS-L system is shown in Fig. 2a.

**Table 1:** GB-SAR measurement parameters used for the campaign.

Parameter	Value	Parameter	Value
Maximum range	500 m	Antenna type	3 (Gain = 19) dBi)
Center wavelength	17.4 mm	Scan length	2 m
Central frequency	17.2 GHz	Acquisition length	368 s
Range resolution	0.8 m	Inter acquisition delay	532 s
Azimuth angle resolution	4.4 mrad	Repeat observation cycle	900 s

A weather station was installed on a pole approximately 2 m from the GB-SAR instrument and 200 m from the slope (Fig. 2). The weather station measured relative humidity, temperature, and pressure every 5 min.

#### In-situ measurements

To verify the results of the GB-SAR system, we installed a displacement meter vertically in the bottom bench of the loess slope (Fig. 2). The measuring range and the accuracy of the displacement meter are 25 mm and 0.02 mm, respectively. The buried depth is 2 m, where the displacement is assumed to be 0 mm. Thus, measured displacement is relative to the position at that depth.

Eight hygrothermographs, which measure soil temperature and moisture, were placed at two sites (site 1 and site 2) on the loess slope (Fig. 1). At each site, four hygrothermographs were placed at depths of 0.1 m, 0.25 m, 0.4 m and 0.6 m, respectively (Fig. 2c). A data acquisition system was placed on the bottom bench of the loess slope to collect the data measured by these sensors (Fig. 2d). The data acquisition system consists of a solar power supply system and data collectors. The data acquisition cycle is 5 min.

A digital elevation model (DEM) is required to interpret the deformation measured by the GB-SAR. The DEM of the slope was generated through photogrammetry from images acquired with an unmanned aerial vehicle (UAV). The original 0.1 m pixel resolution of the DEM was resampled to 0.5 m.

## Methods

### GB-SAR interferometry

For continuous GB-SAR observations, the spatial baseline can be defined as 0 m. Hence, unlike satellite SAR, there is no flat-earth phase. Ignoring thermal noise and other errors, the unwrapped interferometric phase Δφ_21_ for images acquired at times 1 and 2 can be described by^24–26^:

1 $${\Delta \varphi }_{21}={\varphi }_{2}-{\varphi }_{1}={\Delta \varphi }_{0}+{\Delta \varphi }_{dis}+{\Delta \varphi }_{atm}={\Delta \varphi }_{geom}$$

2 $${\Delta \varphi }_{dis}=\frac{4\pi {f}_{c}{\Delta }_{r}}{c}$$

where φ_1_ and φ_2_ are the phase of images acquired at time 1 and time 2, respectively. Δφ_0_ is a measure of the change in the backscatter phase of targets, which can be ignored for persistent targets. Δφ_dis_ is linearly related to displacement Δ_*r*_ along the line-of-sight direction deformation. Δφ_atm_ and Δφ_geom_ are the errors introduced by atmosphere changing and rail moving. We estimated these two errors and corrected for them^21,27,28^. The data preprocessing was done using IBISDV (IBIS Data Viewer) software.

### Heat conduction equation

The temperature of one-dimensional isotropic soil can be described by the classical heat diffusion equation^29^, with the following solution^30^:

3 $$T\left(z,t\right)=\stackrel{-}{T}+{T}_{0}exp\left(-z\sqrt{\omega /2\alpha }\right)\mathrm{sin}\left(wt-z\sqrt{\omega /2\alpha }\right)$$

4 $$\alpha =\lambda /c$$

where *T(z,t)* is the temperature (°C) at time *t* (s) and depth *z* (m), and *z* is positive in the downward direction. α is the apparent thermal diffusivity, *c* and λ are the volumetric heat capacity (J m^-3^ °C^-1^) and the apparent thermal conductivity (W m^-1^ °C^-1^), respectively. *c* and λ may vary with time and depth; for a homogeneous case, they are assumed to be independent of depth and time. The boundary conditions of Eq. (3) are as follows:

5 $$T\left(0,t\right)=\stackrel{-}{T}+{T}_{0}\mathrm{sin}\left(wt\right)$$

6 $$T\left(\infty ,t\right)=\stackrel{-}{T}$$

7 $$T\left(z,0\right)=f\left(z\right)$$

where *T* (0*,t*) is the soil temperature at depth 0 m, and *T* (∞*,t*) is soil temperature when depth tends to infinity. For simplicity, *T* (∞*,t*) is defined as the soil temperature at the depth where the soil temperature does not vary and remains steady at $\stackrel{-}{T}$. *T* (z,*0*) is the soil temperature at the initial time. *f*(*z*) can be a function of depth or a known constant. *T*_0_ is the amplitude of the temperature sine function at the surface, ω = *2π/P* is the radial frequency, and *P* is the period of the fundamental cycle. From Eq. (3), the apparent thermal diffusivity α can be expressed as follows^31^:

8 $$\alpha =\frac{\omega }{2}{\left(\frac{{z}_{2}-{z}_{1}}{ln\left({A}_{1}/{A}_{2}\right)}\right)}^{2}$$

9 $$\alpha =\frac{1}{2\omega }{\left(\frac{{z}_{2}-{z}_{1}}{{t}_{2}-{t}_{1}}\right)}^{2}$$

where *A*_1_ and *A*_2_ are the amplitude of the temperature sine function at depth *z*_1_ and *z*_2_ , respectively; and *t*_1_ and *t*_2_ are the time interval between measured occurrences of maximum soil temperature at depths *z*_1_ and *z*_2_ , respectively. Accurate measurements of *t*_1_ and *t*_2_ are required. The amplitude decreases exponentially with depth, and at a certain depth, it can be estimated by the daily maximum temperature *T*_*max*_ and minimum temperature *T*_*min*_:

10 $$A=\frac{{T}_{max}-{T}_{min}}{2}$$

Additionally, the finite element difference method can be used to solve Eq. (3)^32^:

11 $${T}_{i,j+1}=\alpha \frac{k}{{h}^{2}}{T}_{i+1,j}+\left(1-2\alpha \frac{k}{{h}^{2}}\right){T}_{i,j}+\alpha \frac{k}{{h}^{2}}{T}_{i-1,j}$$

where *T*_*i,j*+1_ is the soil temperature at the *i*th soil layer and (*j* + *i*)th time, *k* is the time interval, and *h* is the depth interval. When α (*k/h*^*2*^) is less than 1/2, the differential scheme is stable and convergent, i.e. the result is reliable.

In this study, we measured soil temperatures at depths of 0.1 m, 0.25 m, 0.4 m, and 0.6 m with the hygrothermographs. In the daily soil temperature simulation, *k* and *h* are equal to 0.5 h and 0.05 m, respectively. The apparent thermal diffusivity is assumed to be 1.98 × 10^–3^ m^2^ h^−1^. According to the soil temperature measurements at the four depths, the daily soil temperature at depth of 0.6 m under fair-weather conditions is stable, which can be defined as the boundary condition (Eq. 6), i.e. $\stackrel{-}{T}$.

The soil temperature simulation is divided into two steps. The first step is to estimate the soil temperature at depth of 0.05 m, which is the boundary condition in the second step (Eq. 5). Thus, soil temperatures are estimated from a depth of 0.1 m to a depth of 0.6 m, and the soil temperatures at the depths of 0.1 m and 0.6 m are the boundary conditions of Eqs. (5) and (6), respectively. The times of the maximum and minimum soil temperature at a depth of 0.05 m are calculated from Eq. (9). The amplitude of the soil temperature at a depth of 0.05 m is calculated from Eqs. (8) and (10). The maximum soil temperature at a depth of 0.05 m is equal to the sum of $\stackrel{-}{T}$ and half of the amplitude of the soil temperature at 0.05 m depth. Similarly, the minimum soil temperature at a depth of 0.05 m is equal to the difference between the two variables. With this procedure, the value and the time of the maximum and the minimum soil temperatures at a depth of 0.05 m are calculated. The second step estimates the soil temperature from a depth of 0.05 m to 0.6 m. The soil temperature at a depth 0.05 m and 0.6 m are the boundary conditions of Eqs. (5) and 6. In the two steps, the boundary condition (Eq. 7) is simulated by the soil temperature at depths 0 m, 0.1 m, 0.25 m, 0.4 m, and 0.6 m at the initial time.

Because the boundary condition (Eq. 7) is a very rough estimate, the preceding results of simulations are not highly accurate and should be excluded. Thus, in daily soil temperature simulations, we exclude the preceding one-day results. According to the measured soil moisture, we cannot assume that soil temperature at a depth of 2 m is constant throughout the year, and thus in the annual soil temperature simulation, the boundary condition (Eq. 6) is not known. The annual soil temperature is not simulated by the heat conduction equation.

### Calculation of heat energy related to thermal deformation

The heat energy exchange within one hour of soil of a specific thickness can be divided into three terms: the energy exchange with the atmosphere across the soil surface, the energy transferred from the previous time intervals, and the energy transferred to the subsequent time intervals. It is assumed that within an hour, the second and third terms are equal at each location. Therefore, the heat energy related to the change in soil temperature and the thermal deformation are assumed equal to the first term, which can be expressed as^33^:

12 $$w={\sum }_{i=1}^{n}{c}_{vi}\Delta {T}_{i}$$

where *w* is the heat energy in J absorbed or released by the soil surface; *C*_*vi*_ and Δ*T*_*i*_ are the volumetric heat capacity (J m^−3^ K^−1^) and the change of temperature of the *i*th layer of moist soil, respectively; and *n* is the number of soil layers. In this study, the soil from 0 to 0.6 m was divided into 12 layers (i.e. *n* = 12). According to previous research, the volumetric heat capacity *C*_*v*_ of the moist soil can be expressed by^34,35^:

13 $${C}_{v}={C}_{w}\theta +{C}_{s}{\rho }_{s}$$

where *C*_*w*_ is the volumetric heat capacity of water, assumed to be 4.20 × 10^6^ J m ^−3^ K^−1^; θ is the volumetric soil water content; *C*_*s*_ is the volumetric heat capacity of dry soil; and ρ_*s*_ is the bulk density of the soil. We measured the volumetric soil water content at depths of 0.1, 0.25, 0.4, and 0.6 m with the hygrothermographs and derived θ from 0 to 0.6 m by linear interpolation. Here, we take *C*_*s*_ to be 0.8 × 10^3^ J kg^−1^ K^−1^ and estimate ρ_*s*_ to be 1.4 × 10^3^ kg m^−3^ for site 1 and 1.52 × 10^n^ kg m^−3^ for site 2 based on laboratory measurements of samples collected at the site.

### Thermal deformation model

In thermal deformation (expansion and contraction), the change in length Δ*L* of a sample of loess material caused by a temperature change can be expressed by:

14 $$\Delta L=\beta \Delta TL$$

where *L* (m) is the initial length of the sample at room temperature, Δ*T* (°C) is the change in temperature, and β (°C^−1^) is the linear thermal expansion coefficient of the sample. In this paper, the temperature of the loess varies with depth, therefore the expansion and contraction Δ*L* of the whole slope caused by the temperature change can be derived as.

15 $$\Delta L={\sum }_{1}^{n}\beta \Delta {T}_{i}{L}_{i}$$

where *L*_*i*_ is the initial thickness of the *i*th loess layer, and Δ*T* is the change in temperature of the ith loess layer. β is the linear thermal expansion coefficient of loess, and in this study, is independent of temperature. As only the daily soil temperature is available from the heat conduction equation, we simulated the thermal deformation in the daily deformation cycles. The loess soil from a depth of 0.1 m to a depth of 0.6 m is divided into 12 layers, each 0.05 m thick. The linear expansion coefficient β of common materials is about 10^–6^ ~ 10^–5^ °C^−1^^36^, The linear thermal expansion coefficient of loess is approximately 35 × 10^–5^ °C^−1^^37,38^.

## Results and discussion

### Observed slope deformation

#### Daily observations

**Figure 3:**
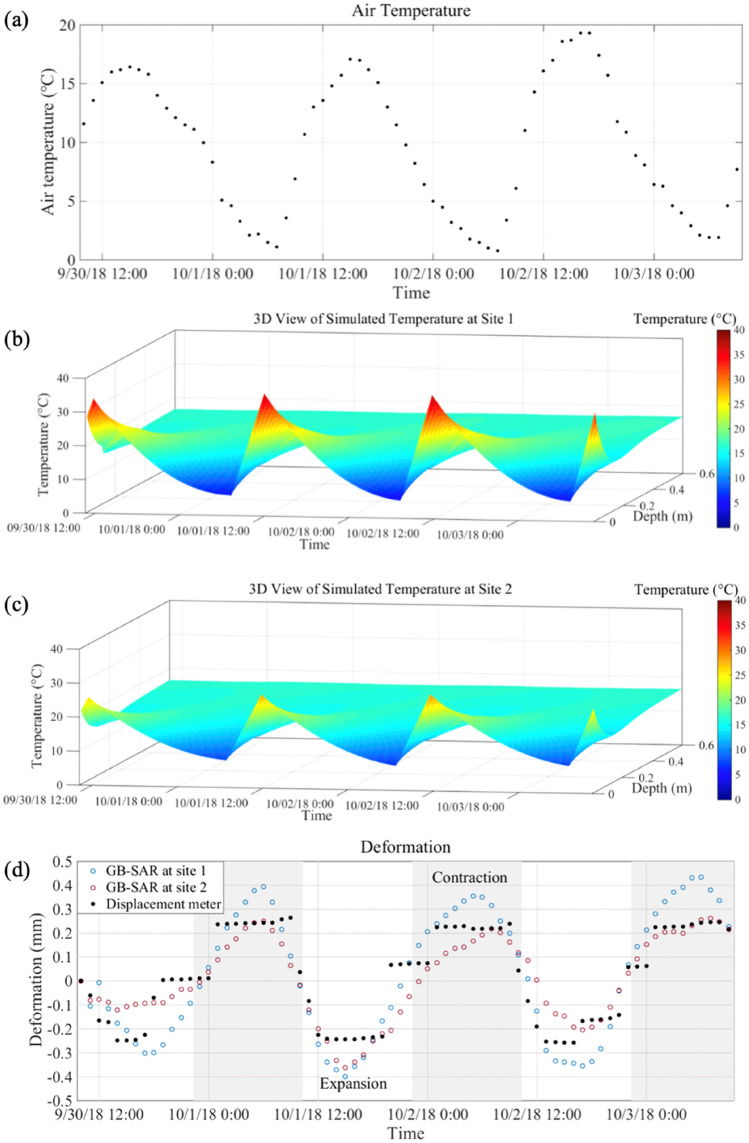
The daily cycle of temperature and deformation over three days from 10:15 on 30 September to 10:14 on 3 October 2018. (**a**) Air temperature. (**b**) and (**c**) 3D view of soil temperature at sites 1 and 2. (**d**) Comparison of deformation measured by the GB-SAR sensor and the displacement meter. Negative and positive values indicate deformation toward and away from the GB-SAR sensor, respectively. Temperature and deformation fluctuation appear to be highly correlated. Air temperature and deformation were sampled every hour.

We show daily observations of slope deformation for a three-day period (30 September to 3 October 2019) in Fig. 3. Measured air temperature and simulated soil temperature with depth are also shown in this figure. The simulated soil temperatures are derived from hydrothermograph measurements and heat conduction equations, as described above.

Air temperature cycles (Fig. 3a) show a minimum temperature near 1 °C, reached at about 7:00, and a maximum temperature near 17 °C, reached at about 15:00. The air heats up faster than it cools down at the study area. Similarly, soil temperatures exhibit cyclic fluctuations, which is attributed to the changing solar radiation through the day (Fig. 3b,c). Temperatures of the shallow soil are similar to air temperatures. The times of maximum and the minimum soil temperatures increasingly lag air temperature extremes due to conduction of heat in the soil. The amplitude of the soil temperature cycle decreases with depth, and at depths greater than 0.5 m there are no measurable changes in soil temperature.

Deformation measured by the GB-SAR sensor is consistent with that recorded by the displacement meter (Fig. 3d) and validates the cyclic patterns described above. The deformation measured by the GB-SAR sensor shows a distinct, somewhat smooth sinusoidal pattern. The displacement meter data show an episodic contraction and expansion. The overall pattern of the two sets of deformation measurement is similar, and the differences between them are generally less than 0.1 mm. The GB-SAR deformation data are assumed to be representative of the slope behavior and adopted for further analysis.

Maximum contraction generally happened between approximately 3:00 and 8:00, and the maximum expansion occurred between 14:00 and 17:30. Between the times of maximum expansion and contraction, the surface “breathing” is relatively continuous. The expansion period is defined as the interval when the surface expands relative to the average position, typically between 10:30 and 22:30 (the white background in Fig. 3d). The contraction period is the interval when the surface contracts relative to the average position, typically from 22:30 to 10:30 (the gray background in Fig. 3d). Deformation measured by GB-SAR and soil temperatures appear highly correlated, showing that soil expansion and contraction mainly follow the cyclic fluctuations of soil temperature between 0 m (surface) and approximately 0.5 m depth into the slope.

**Figure 4:**
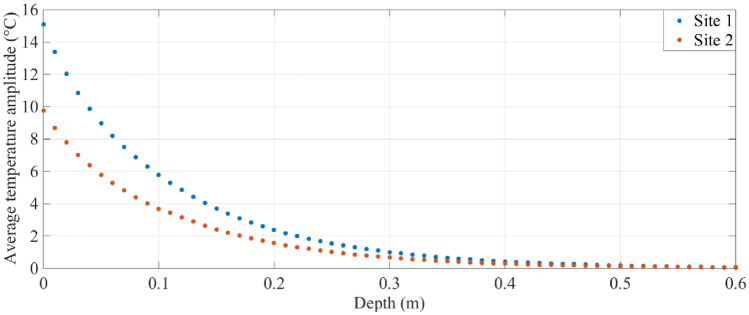
Average soil temperature from 0 to 0.6 m depth at sites 1 and 2 over a three-day period.

Figure 3d shows that the maximum contraction and expansion at site 1 are larger than those at site 2. The differences between the two sites stem from the fact that the soil temperature fluctuations at site 1 are larger than those at site 2. Figure 4 shows the average soil temperatures as a function of depth at the two sites over the same three days as in Fig. 3. It is clear from the data that the temperature changes at depths less than about 0.3 m are larger at site 1 than site 2. The maximum difference between the two sites is > 5 °C at a depth of 0 m, decreasing to approximately 0 °C at depths > 0.3 m. We attribute these differences to differences in soil moisture at the two sites.

**Figure 5:**
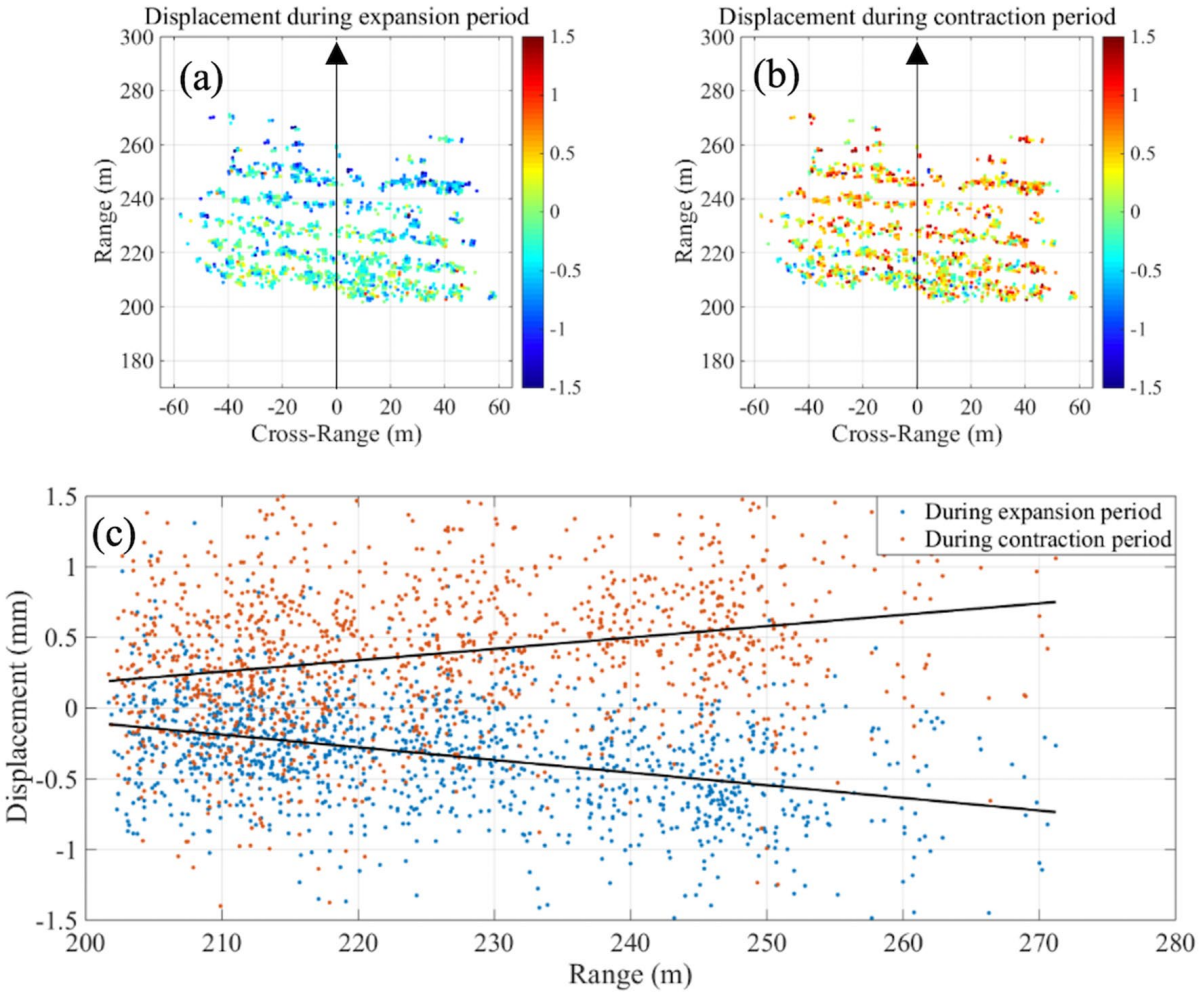
Slope displacements measured by the GB-SAR during (**a**) the maximum expansion period (16:14 on 30 September 2018), and (**b**) the maximum contraction period (07:29 on 3 October 2018). (**a**) and (**b**) show the displacement maps, where range is the distance from the GB-SAR (lower range corresponds to the toe of the slope, higher range corresponds to the crest, and cross-range is in the direction of the width of the slope, zeroed at the location of the radar). (**c**) Displacement plotted against range with dark lines showing trends during the expansion/contraction periods, i.e. increasing cyclic displacements near the crest compared to the toe of the slope.

Spatially, the amplitudes of the difference between cyclic expansion and contraction increase linearly from the bottom of the slope to the top (Fig. 5). To describe this spatial variation, Fig. 5 shows the surface deformation at the times of maximum expansion (16:14 on 30 September 2018) and maximum contraction (07:29 on 3 October 2018). The information is presented as a map of surface deformation from the perspective of the radar, where range is the distance from the GB-SAR (lower range corresponds to the toe of the slope, higher range corresponds to the crest) and cross-range is in the direction of the width of the slope, zeroed at the location of the radar, and plotted against range (Fig. 5c). The graphs in Fig. 5 are in GB-SAR coordinates, with the location of the GB-SAR sensor defined as the origin. The direction of the GB-SAR linear rail is the cross-range direction. The direction perpendicular to the rail and along line-of-sight direction is defined as the range direction. At the bottom of the slope (range in Fig. 5a,b = 201 m), the expansion and the contraction each average about 0.15 mm. At the top of the slope (range 270 m in Fig. 5a,b), the expansion and the contraction average approximately 0.7 mm. The measured expansion has the same magnitude of the contraction, suggesting that the deformation is recovered and in the elastic regime.

#### Annual observations

**Figure 6:**
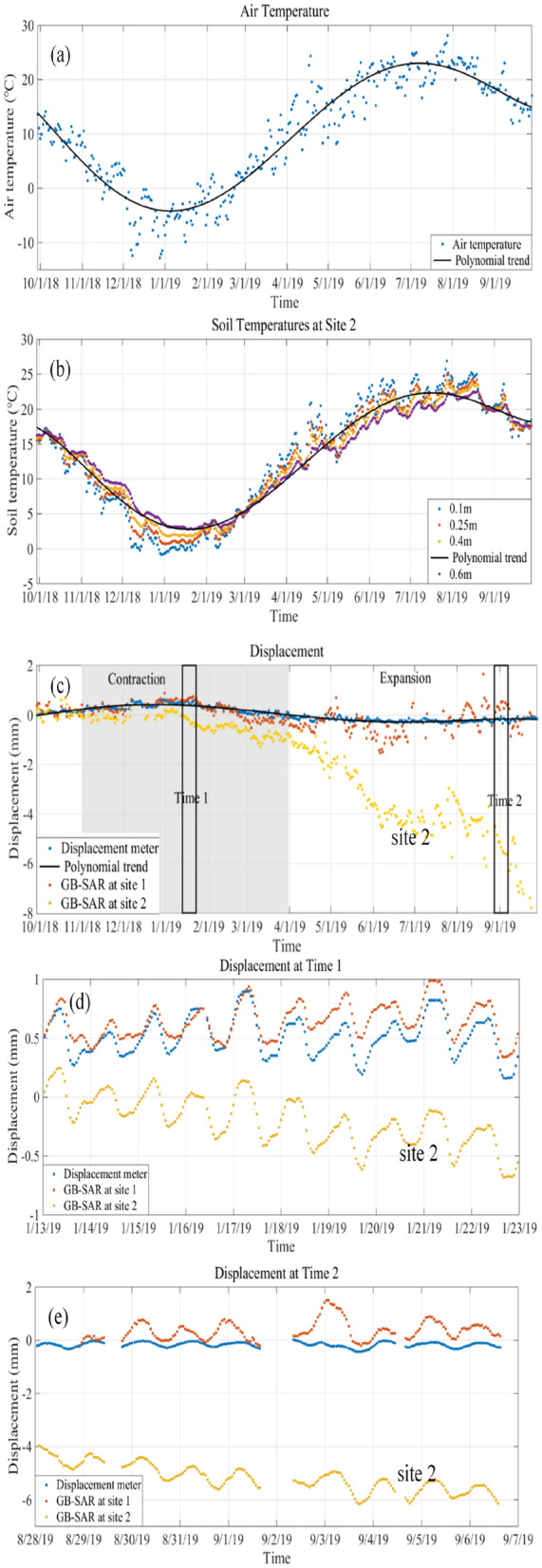
The annual cycle of air and loess temperature, and slope expansion/contraction between 30 September 2018 and 1 October 2019. (**a**) Daily average air temperatures (dots) and their polynomial trend (black line). (**b**) Daily average soil temperatures (dots) at site 2 and their polynomial trend (black line) at four depths. (**c**) Daily deformation measured at sites 1 and 2 with GB-SAR sensor and the displacement meter (black line is the polynomial trend for the displacement meter). (**d**) and (**e**) Deformation measured by the displacement meter and GB-SAR sensor between the 13 and 23 January 2019 and between August 28 and 17 September 2019. Negative and positive displacement values indicate movement towards and away from the GB-SAR sensor. Observations suggest a close correlation between air temperature and slope expansion/contraction at the annual scale.

The annual cycles of daily average air and soil temperatures and slope expansion/contraction between 30 September 2018 and 1 October 2019 are shown in Fig. 6. Soil temperature differences between the two sites are not significant compared to their fluctuations throughout the year, thus we only show data from site 2 in Fig. 6b. The minimum air temperature at the study site, about − 5 °C, occurs in mid to late January; the maximum of 23 °C is in late July to early August (Fig. 6b). As in the case of the annual cycles, the times of the maximum and the minimum soil temperatures show a lag that increases with soil depth. Maximum and minimum soil temperatures also decrease with depth, showing less variability as depth increases.

The annual cyclic contraction and expansion at site 1shows that deformations are recoverable, therefore suggesting that no damage has occurred to the loess material and that the material strength is preserved. The maximum contraction occurs approximately in mid to late January, and maximum expansion in late July and early August. The amplitude of the average annual fluctuation is about 1 mm. The contraction period typically extends from mid-November to mid-April of the following year (gray background in Fig. 9), and the expansion period is from mid-April to mid-November (the white background in Fig. 9).

The annual cyclic deformation at site 2 shows that deformation towards the GB-SAR (out of slope) is not fully recovered (Fig. 6c). The unrecovered cumulative deformation increases through the period. Figure 6d,e show the same pattern –the daily cycles at site 2 have expansions that are not recovered during the contraction period, both in cold and warm weather. This suggests that damage has occurred and that the material strength has been reduced.

**Figure 7:**
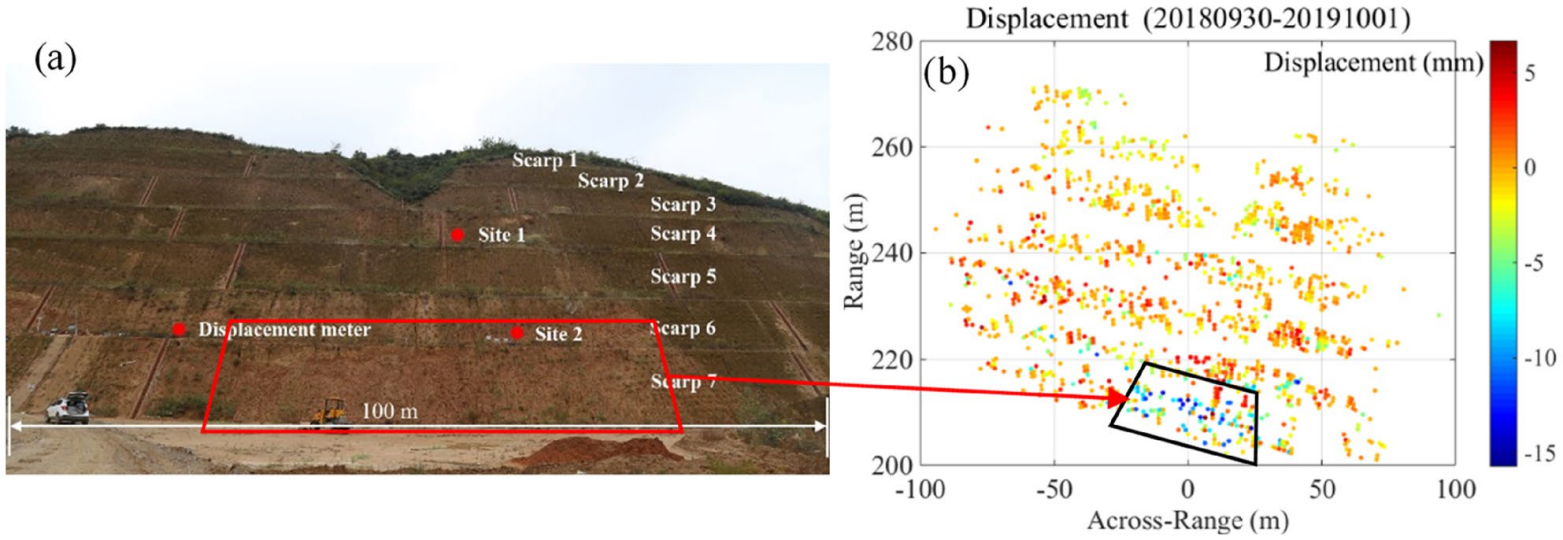
Slope deformation between 30 September 2018 and 1 October 2019. The red (**a**) and black (**b**) rectangles indicate the area showing non-recoverable deformation. Negative values are out-of-slope deformation.

Figure 7 shows the deformation between 30 September 2018 and 1 October 2019. The red rectangle in Fig. 7a and the black rectangle in Fig. 7b rectangle indicate the area of the slope where measured deformations were not fully recovered, with approximately 8 to14 mm of out-of-slope deformation in one year, sufficient to cause small-scale landslides^39^.

#### Soil moisture

**Figure 8:**
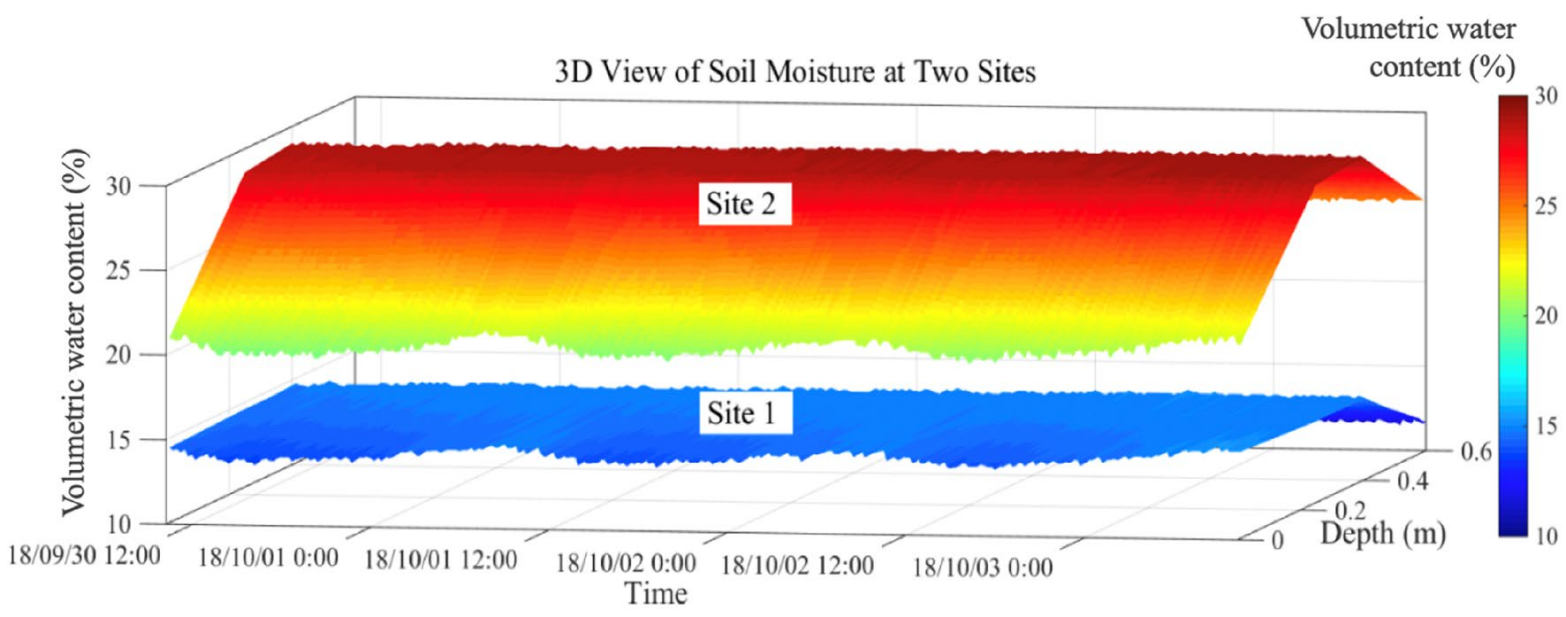
3D view of soil volumetric water content from 0 to 0.6 m depth at sites 1 and 2 over a three-day period. Soil moisture contents at depths of 0.1, 0.25, 0.4, and 0.6 m were measured with hygrothermographs; values at other depths were calculated by linear interpolation.

The soil volumetric water content at site 2 is consistently greater than that site 1 (11–15% at site 1, and 20–30% at site 2) (Fig. 8). Rainfall infiltration is the major source of soil moisture at the study site. Low infiltration due to the high slope angle (approximately 48°) and consequent high surface runoff result in relatively low soil moisture at the top of the slope. Moisture is also lost at the top of the slope due to exposure to wind. Rainwater infiltrates the lower part of the slope and ground water collects there, leading to higher soil moisture^40–42^.

Despite these differences, soil moisture at the two sites has some similarities. The soil moisture at both sites initially increases with depth and then decreases (Fig. 8). Water contents at both sites show the same diurnal fluctuations, which are mainly the result of evapotranspiration. Soil moisture begins to decline at approximately 8:00 as the sun rises and evapotranspiration increases. In the late afternoon, around 16:30, evaporation decreases as the slope is initially shadowed and the sun then sets, resulting in a small increase in soil moisture.

### Mechanism of cyclic deformation of the loess slope

We propose that the observed correspondence between temperature fluctuation and slope cyclic deformation reflects the response of the soil mass to temperature fluctuations at shallow depths, i.e. expansion and contraction of the loess grains. The amount of expansion and contraction depends on the soil temperature variations caused by fluctuations in air temperature, the thermal characteristics of loess grains, and the soil moisture content. This relationship can be quantified in terms of heat energy and energy transfers between the air and the soil, and through the soil mass.

**Figure 9:**
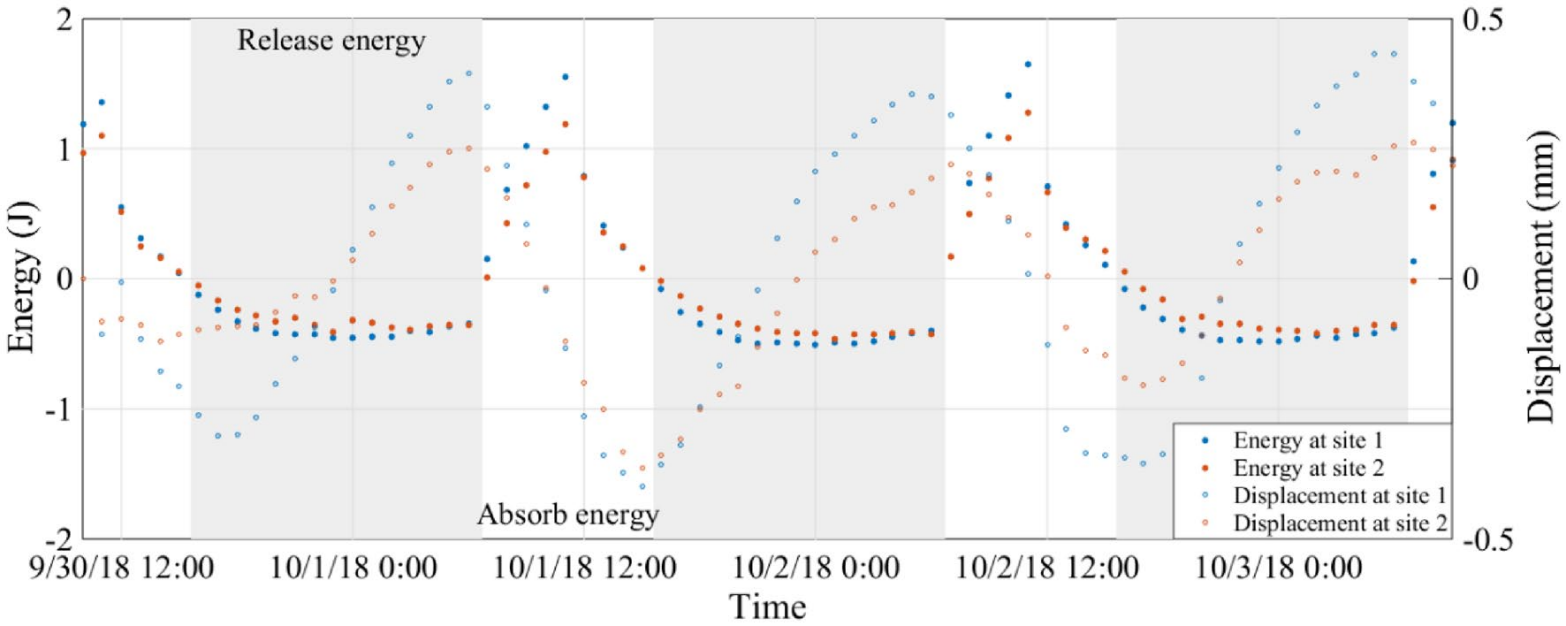
Energy fluctuation at the two sites during a three-day interval, estimated from soil temperature and moisture data. Positive values indicate energy absorption (white background), and negative values indicate energy release (gray background).

The heat energy at the two sites was calculated based on soil temperatures and moisture contents, and is shown in Fig. 9. As expected, the energy has a cyclic daily fluctuation, which corresponds to energy transfers between the soil and the air. As the sun rises (at 6:40 during the three-day interval), solar radiation begins to increase and air temperature increases, soon becoming higher than that of the soil surface. At about 7:00, energy begins to transfer from the atmosphere to the soil surface, the soil absorbs energy, and the energy flux turns positive. At this moment, the soil temperature begins to increase, the amplitudes of atomic vibration in the soil increase, and the average separation of the atoms increases, leading to volumetric change of the water and soil grains. As a result, the loess deforms out of slope^43^. In the afternoon, as the sun lowers toward the horizon, solar radiation begins to decrease, and at approximately 15:00, the temperature of the air decreases and approaches equilibrium with the soil surface. As air temperature continues to decrease, energy begins to transfer from the soil surface towards the atmosphere, the soil releases energy, and the energy flux becomes negative. From the time of maximum contraction to maximum expansion, the slope absorbs energy, and from the time of maximum expansion to maximum contraction, the slope releases energy. In a daily cycle, the total values of absorbed and released energy are generally equal; therefore energy is generally conserved during the daily “breathing” of the slope except for minor temperature differences from day to day.

We observed that the absolute calculated value of the energy at site 1 is sometimes larger (up to 19%) than that at site 2. This difference possibly results from two factors. First, there is a difference in solar radiation at the two sites, where direct sunlight reaches the crest of the slope sooner than the toe. This difference was observed in the temperature fluctuation and changes with depth at the two sites. Second, soil moisture content is also different at the two sites, therefore yielding a different energy transfer. Differences in soil temperature between sites are likely controlled by differences in heat capacity, which in turn are mainly controlled by differences in soil moisture.

The amplitude of soil temperature change increases as the soil moisture decreases. Specifically, because soil moisture at site 2 is higher than that of site 1, soil temperature changes at site 2 are lower than at site 1. The spatial variation of soil moisture in the slope would have a substantial effect on the amount of energy transferred and therefore will have a marked influence in the spatial variation of soil temperature and its manifestation in cyclic deformation. Soil moisture at site 2 is higher than that of site 1, leading to a higher heat capacity at the former site than the latter. Consequently, soil temperature fluctuations at site 1 are larger than those at site 2.

In our study, the hygrothermographs were installed at four depths (0.1, 0.25, 0.4, and 0.6 m), thus soil moisture at depths > 0.6 m is unknown. Accordingly, a linear interpolation of soil moisture from 0.6 m to 2 m would not be reliable. Therefore, we have not attempted to quantify energy changes on an annual basis. However, it is understood that daily and annual cyclic deformations follow the same mechanism, therefore the slope absorbs energy from the time of maximum contraction to maximum expansion and releases energy and from the time of maximum expansion to the maximum contraction within each annual cycle.

### Forecasting annual and daily deformation cycles, and what to expect with climate change

**Figure 10:**
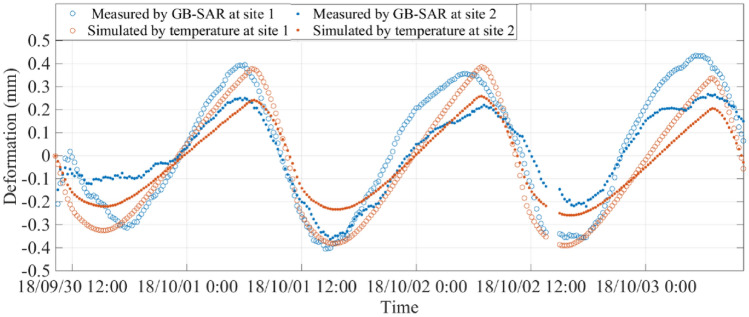
Deformation measured by the GB-SAR sensor at sites 1 and 2, and simulated deformation based on thermal deformation model and temperature measurements.

The close relation between temperature fluctuations and the slope deformation cycle allows us to postulate a predictive deformation model for the loess slope. The daily deformation cycles measured by GB-SAR and simulated by temperature at the two sites between 30 September and 3 October 2019 are shown in Fig. 10. The agreement between the measured and simulated daily deformation cycle supports the postulated mechanism and the infdataluence of moisture and soil temperature at depths up to 0.6 m. However, we do not have soil temperature throughout the year and thus cannot simulate the soil temperature and thermal deformation in annual cycles.

**Figure 11:**
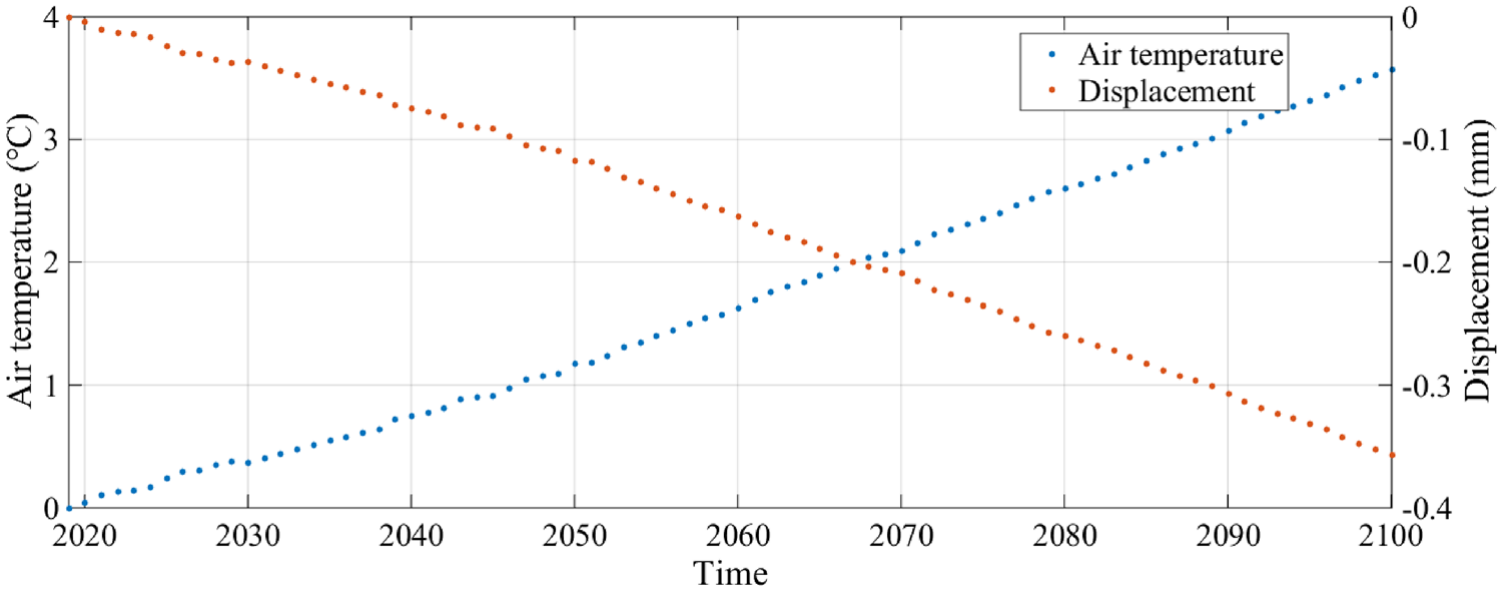
Projected air temperature and deformation of the loess slope on 15 January of each year from 2020 to 2100, referenced to 2020. Negative values indicate deformation toward the GB-SAR sensor (expansion of the slope). The loess slope expands to 0.35 mm (average, without considering annual and daily breathing cycles) as atmosphere temperature increases 3.5 °C.

The predictive model also provides a tool to explore the potential effects of climate change on the cyclic deformation of the loess slope. Temperature extremes are difficult to predict, but a first approximation of the effect of warmer climate on the loess slope can be made by considering the maximum amplitude of slope deformation observed in the present-day daily cycles (about 1 mm) to that expected for average temperatures in the future. By the end of this century, assuming the IPCC high-emission scenario RCP (representative concentration pathway) 8.5, the global average temperature will be 3.5 °C higher than today^44^. Given that energy rapidly transfers between the atmosphere and the soil, we assume the surface soil temperature, on average, will increase by the same amount. The heat conduction equation can be used to calculate future soil temperatures with depth in the loess slope in that we have studied, assuming a linear increase in average air temperature increase of 3.5 °C between now and 2020. Figure 11 shows temperature and deformation for the time on January 15 of every year from 2020 to 2100 when the air temperature is at a minimum and the contraction of the slope is at a maximum in the annual cycle. Because we are calculating the relative increase in average slope expansion due to the average increase in temperature, the choice of the time of year does not affect the calculations and conclusions. Figure 11 shows that with an assumed increase in air temperature of 3.5 °C from 2020 to 2100, the loess slope will expand an average of 0.35 mm. This represents a 70% increase in expansion during the daily cycle in comparison to the present deformation. As the air temperature increases, the increasing transfer of energy from the atmosphere to the soil will lead to a substantial increase in deformation, which will contribute to an accelerated thermal fatigue of the loess forming the slope. This could lead to accelerated deterioration of the shallow layers of the loess slope and failure due to gravitational forces.

## Conclusions

We have documented the spatio-temporal characteristics of cyclic expansion and contraction of a loess slope near Yan’an, in Shanxi Province, northwestern China using GB-SAR technology. The mechanism of deformation was analyzed using empirical and physical models of soil temperature and moisture. Our conclusions are as follows:

(1) The loess slope shows daily and annual cyclic expansion and contraction, which mainly result from the cyclic fluctuation of soil temperature.

(2) The cyclic expansion and contraction are caused by energy transfers. When the slope moves outward, it absorbs energy, and when the slope moves inward, it releases energy. This is reflected in volumetric changes of the water within the soil and differences in excitation of the soil particles.

(3) Soil volume changes are associated with stress changes in the soil. Fully recovered deformations between cycles suggest that deformations are within the elastic regime of the loess material. Non-recoverable deformations, observed at one site on an annual timescale, suggest damage of the loess (breakage of bonds between soil grains) and therefore a loss in soil strength.

(4) Cyclic expansion and contraction characteristics of a loess slope can be used to evaluate damage in the soil materials that could lead to surficial slope failures.

(5) Increases in temperature predicted by some climate change models suggest that cyclic deformations of the loess slope at our study site might increase by approximately 70%.
